# Assessment of genomic prediction capabilities of transcriptome data in a barley multi-parent RIL population

**DOI:** 10.21203/rs.3.rs-6145169/v1

**Published:** 2025-03-31

**Authors:** Christopher Arlt, Delphine van Inghelandt, Jinquan Li, Benjamin Stich

## Abstract

The field of genomic selection (GS) is advancing rapidly on many fronts including the utilization of multi-omics datasets with the goal to increase prediction ability (PA) and to become an integral part of an increasing number of breeding programs ensuring future food security. In this study, we used RNA sequencing (RNA-Seq) data to perform genomic prediction (GP) on three related barley RIL populations investigating the potential of increasing PA by combining genomic and transcriptomic datasets, adding whole genome sequencing (WGS) SNP data, functional parameter filtering, and empirical quality filtering. Our RNA-Seq data were generated cost-efficiently using small footprint plant cultivation, high-throughput RNA extraction, and library preparation miniaturization. We also examined the depth of the sequencing as an additional cost-saving measure. We used five-fold cross-validation to evaluate the PA of the gene expression dataset, the RNA-Seq SNP dataset, and the consensus SNP dataset between the RNA-Seq and parental WGS data, resulting in PAs between 0.73 and 0.78. The consensus SNP dataset performed best, with five out of eight traits performing significantly better compared to a 50K SNP array, which served as a benchmark. The advantage of the consensus SNP dataset was most prominent in the inter-population predictions, in which the training- and validation-set originated from different RIL sub-populations. We could therefore not only show that RNA-Seq data alone are able to predict various complex traits in barley using RIL, but also that the performance can be further increased by WGS data for which the public availability will steadily increase.

## INTRODUCTION

The continuous increase in the global population and the per capita crop demand create the need for agricultural expansion and advancements in plant breeding (Lenaerts et al., 2019; Tilman et al., 2011). The ecological impact of land clearing due to agricultural intensification is already substantial and will increase further (Zabel et al., 2019; Burney et al., 2010). This makes improving crop yield one of the most important tasks (Burgess et al., 2023).

The improvement of agriculturally important quantitative traits by plant and animal breeding was achieved for thousands of years without knowledge of genetics principles but simply based on artificial human selection on phenotypes (Purugganan and Fuller, 2009; Pourkheirandish and Komatsuda, 2007; Wright, 2005). This changed in recent decades with the increase in knowledge and capabilities in genetics, which in turn was possible by dramatic progress on genotyping and sequencing approaches. This enabled the development and application of marker-assisted selection (MAS) (Dekkers and Hospital, 2002; Lande and Thompson, 1990; Fernando and Grossman, 1989). The challenge is that most agriculturally relevant traits are quantitative traits controlled by many genes with each having only a small effect (Glazier et al., 2002; Mackay, 2001), while MAS is most effective when large-effect loci contribute to the trait of interest (Heffner et al., 2009). With the increasing availability of high density genome-wide genetic marker data, genomic selection (GS) was introduced as a method to estimate breeding values using all available marker data (Meuwissen et al., 2001) and not only those that were significantly associated with the trait. GS alleviated the downside of classical MAS when trying to predict complex quantitative traits with many small-effect loci (Zhao et al., 2014) and increased the rate of genetic gain. While GS was first studied in the context of animal breeding, it was later adopted by plant breeders (Heffner et al., 2009; Zhong et al., 2009; Bernardo and Yu, 2007). The reduction in costs to produce marker data using next-generation sequencing techniques such as genotyping by sequencing (GBS) further increased the popularity of GS (Bhat et al., 2016).

Over the last decade, advances in GS methods have led to increased prediction abilities. For example, utilizing high-throughput phenotyping data as predictors increased the performance of multivariate GS models (Rutkoski et al., 2016). Additionally, traditional GS models were expanded to multi-trait GS models (Tsai et al., 2020; Lyra et al., 2017; Jia and Jannink, 2012). Furthermore, multi-environmental GS models increased the predictability in multiple studies (Hu et al., 2023; Li et al., 2019). The most recent advances are the inclusion of deep learning, machine learning, and artificial intelligence in the GS workflow (Sandhu et al., 2021; Montesinos-Lopez et al., 2021; Bayer et al., 2021; Washburn et al., 2020; Harfouche et al., 2019). This is an ongoing field of research and is not yet fully explored, with studies showing limitations or at least the need for adjustments to current implementations (Ubbens et al., 2021).

While the core of most GS models is a relationship matrix derived from genomic marker data, in the field of multi-omic GS additional sources of information like transcriptome and metabolome data can be used as predictors. The transcriptome is a promising predictor, bridging the gap between the genome and the trait (Azodi et al., 2020). Quantified gene expression can be captured using a microarray or mRNA sequencing (RNA-Seq). RNA-Seq data are more versatile than microarrays as from such data not only gene expression information can be extracted but also sequence variants in the portion of the genome covered by the sequenced RNA (Azodi et al., 2020; Weisweiler et al., 2019). Because both aspects are rarely considered together and to our knowledge no research is available that evaluates functional or quality based sub-setting of sequence variants from RNA-Seq data, the full potential of RNA-Seq datasets for GS is not yet sufficiently studied.

Within the field of plant breeding, several studies have previously shown that including transcriptome and metabolome data (multi-comics) in GS models has the potential to increase prediction capabilities. For example, Michel et al. (2021) added incomplete RNA-Seq data to complete genomic marker data to assess disease resistance phenotypes in wheat. In addition, a multi-omics prediction study in oat used transcriptomic and metabolomic data to compare single-environmental trials and multi-environmental trials (Hu et al., 2021). Guo et al. (2016) was able to successfully combine transcriptomic and metabolomic data with genomic markers from diverse maize inbred lines to increase predictability in GS. Data using multi-omics GS in barley, on the other hand, are extremely limited and only recently started to emerge (Wu et al., 2022).

Almost all previous studies focused on diversity panels to validate the capabilities of their GS model (Hu et al., 2021; Michel et al., 2021; Westhues et al., 2019; Schrag et al., 2018; Westhues et al., 2017; Guo et al., 2016). However, in plant breeding programs, half-sib or full-sib families are typically used from which the most appropriate progenies are selected. Nevertheless, to the best of our knowledge, no earlier study evaluated the potential of multi-omic GS models in this context.

In this study, we explore the capabilities of low-cost RNA-Seq data to perform genomic prediction (GP) on three connected spring barley RIL populations with 237 individual lines for eight agriculturally important traits each measured in up to seven environments. Based on this dataset, the potential to increase the performance of the GP model by combining genomic and transcriptomic data, functional parameter filtering, and a novel empirical two-step quality filtering approach is evaluated. Lastly, we examine multiple optimization parameters that could lead to cost and time savings without sacrificing prediction ability.

## MATERIALS AND METHODS

### Genetic material

The HvDRR population was developed from pairwise crosses among 23 diverse parental inbreds (Weisweiler et al., 2019) using the double round-robin (DRR) mating design (Stich, 2009). Our study was based on 237 recombinant inbred lines (RIL) from three HvDRR sub-populations (Casale et al., 2022) that were derived from pairwise crosses among parental inbreds Spratt-Archer, HOR8160, and Unumli-Arpa (Fig. 1). The three sub-populations were designated in the following as HvDRR13 (65 RIL), HvDRR27 (92 RIL), and HvDRR28 (80 RIL).

### Plant cultivation for RNA extraction

All RIL were cultivated in a randomized augmented incomplete block design. A block consisted of 24 samples, including 21 RILs and all three parents as controls (Fig. 1). The cultivation workflow was identical to that described previously (Arlt et al., 2023). In summary, for each RIL, 15 seedlings were cultivated in vertically stacked square Petri dishes for seven days in a reach-in growth chamber under the following conditions: 70% relative humidity, 16 hours of light (6:00 - 22:00), 22 degrees (day) / 20 degrees (night), and light intensity about 400 $\mu mol\ m^{-2}\ s^{-1}$ (Fig. S1). The time of day for planting and harvesting were similar (within two hours) for all samples. All plants in the same block were processed simultaneously.

### RNA extraction

The seedlings were harvested as a whole, immediately frozen, and ground. From 50 mg of plant material, total RNA was extracted using TRIzol reagent (Thermo Fisher, USA). The manufacturer's protocol was adapted as described below to fit a

96-well format and to use less reagent (Arlt et al., 2023). The input plant material and all the reagents for extraction were halved. The final washing step in 75% ethanol was repeated one additional time to ensure that all the remaining phenol was removed. All other steps were performed as proposed by the manufacturer. The total RNA concentration was quantified using a NanoPhotometer NP 80 from Implen (Germany). A total of 33 extractions were randomly selected for evaluation using the Fragment Analyzer (Agilent, USA).

### Library preparation

The mRNA was selected based on a poly-A tail mRNA capture method (Vazyme, China) using $1\mu g$ total RNA input. The full-length mRNA library was constructed using the VAHTS Universal V6 RNA-seq Library Prep Kit for Illumina kit from Vazyme (China). We miniaturized the procedure by reducing the volume of reagent to 25% of the original amount (Arlt et al., 2023). Size selection and cleanup was performed using magnetic DNA Clean Beads (Vazyme, China). Apart from the miniaturization, the manufacturer's protocol was followed aiming for 250-450bp long inserts.

### Sequencing and read processing

Sequencing was performed by BGI on the DNBSEQ-G400 platform. All 284 samples were pooled and a total of 3.70 billion 150 bp paired end reads were sequenced with an average of 13.0 million read pairs per sample (Arlt and Stich, 2022). Various quality statistics of raw sequencing reads were evaluated using fastQC (Andrews, 2019) and afterwards trimmed with trimmomatic (ILLUMINACLIP:TruSeq3-PE:2:30:10:1:TRUE SLIDINGWINDOW:4:15 LEADING:3 TRAILING:3 MINLEN:36) (Bolger et al., 2014). The trimmed reads were then aligned to the Morex V3 reference sequence (Mascher, 2019) using Hisat2 (–no-softclip –max-seeds 1000) (Kim et al., 2019).

### Sequence variant calling

Variant calling was performed using the following three datasets: RNA sequencing, SNP array, and whole genome sequencing (WGS) data. (1) For all RNA-Seq datasets, variant calling was performed using the bcftools mpileup (filter: -q 20 -Q 20) and call function (Li et al., 2009). All duplicated genotypes were united using the major allele as consensus. All variants with missing parental genotype information were excluded. Afterwards, the data was cleaned by setting all heterozygous alleles to NA, due to the near complete homozygosity of all inbred lines used in this study. Additionally, all RIL alleles inconsistent with parental alleles were set to NA. The missing data was median imputed for each RIL population independently. The resulting dataset was designated in the following as $SNP^{Total}_{RNAseq}$. To test the impact of quality filtering on a less strictly cleaned dataset $SNP^{Raw}_{RNAseq}$ was created, which did not set heterozygous / inconsistent allele calls to NA and did not remove variants with missing parental data. (2) Already existing SNP array data for the same RIL were included in this study ($SNP^{Total}_{Array}$). The data were generated using the Illumina 50K iSelect SNP array for barley (Bayer et al., 2017). The SNP array dataset was filtered as described by Casale et al. (2022). (3) Additionally, all available RNA-Seq SNP were intersected with WGS SNP data from the parental inbreds (Weisweiler et al., 2022), selecting only variants that were present in both datasets and therefore building a consensus subset ($SNP^{Total}_{WGS}$). For $SNP^{Total}_{WGS}$, missing SNP data were imputed using parental WGS SNP data with Beagle (Browning et al., 2021). For all datasets described above, genetic differentiation $G_{st}$ was calculated according to Nei (1973).

### RNA-Seq SNP function filtering

The function of the SNP in the $SNP^{Total}_{RNAseq}$ dataset was determined using the SnpSift and SnpEff programs (Cingolani et al., 2012a,b). We used the SNP functional information to filter the total dataset and create two new subsets. The first SNP functional subset contained only SNP within the 5’UTR and 3’UTR within a 5 kb distance to the coding region (SnpSift.jar filter ”(ANN[*].EFFECT has ’upstream_gene_variant’) || (ANN[*].EFFECT has ’downstream_gene_variant’)”) and was designated in the following as $SNP^{Reg.}_{RNAseq}$. The second SNP function subset excluded all synonymous SNP, only selecting missense variant SNP in the coding region (SnpSift.jar filter ”ANN[*].EFFECT has ’missense_variant’”) and was designated in the following as $SNP^{CDS}_{RNAseq}$. Missing data was median imputed and all monomorphic markers were excluded.

### RNA-Seq SNP quality filtering

The following quality filtering parameters were used to filter $SNP^{Total}_{RNAseq}$: read depth (DP), minor allele frequency (MAF), quality score (QUAL), and number of samples without data (NS). The filtering was based on the information from the vcf file. After determining the minimum and maximum values of $SNP^{Total}_{RNAseq}$ for the four criteria, the full spectrum was divided into 21 segments based on relative filtering strength (from 0% to 100% in 5% steps). For each step, the results of the genomic prediction cross-validation were analyzed to determine the relative filter strength that performs best for each criterion.

In a last step, the subsets with the best filtering performances were combined creating the inclusive marker intersects, which were evaluated for their prediction ability. The results were compared to a standard SNP filtering procedure (missing rate < 20%, MAF > 0.05) (Atanda et al., 2022; Wen et al., 2018) and the prediction abilities were tested for their significance using pairwise t-tests. The best performing combination was selected and the data were cleaned and imputed analogously to the procedure of $SNP^{Total}_{RNAseq}$. The resulting quality-filtered RNA-Seq SNP subset was designated in the following as $SNP^{QC}_{RNAseq}$.

### Read count calculation

The expression per transcript was determined for all samples with the help of ht-seq count (–mode union) (Anders et al., 2015). The Trimmed Mean of the M-values (TMM) method was used to apply a between-sample normalization using the R package edgeR (Robinson and Oshlack, 2010). All transcripts in which at least 2% of the samples had non-zero read counts were included in the total expression dataset. The original read counts ($GE^{Total}_{RNAseq}$) were filtered using a counts per million (cpm) threshold which maximizes the number of differentially expressed genes (DEG) resulting in the filtered expression dataset ($GE^{Filter.}_{RNAseq}$). The method used is similar to the DEseq2 filtering approach (Love et al., 2014). DEG was calculated for each of the available genotypes (all RILs of the three populations and the parental inbreds) and compared to the general mean. The dispersion was estimated by edgeR based on the replicated parental inbreds. An additional dataset was created that included only the DEG ($GE^{DEG}_{RNAseq}$). For all expression subsets, a per transcript normalization was applied to correct for any block effects on the expression levels. We used the following linear mixed model for each transcript: $y_{ijk} = \mu + G_i + B_j + \varepsilon_{ijk}$, with the genotype effect ($G_i$, fixed), block effect ($B_j$, random), and an error term ($\varepsilon_{ijk}$). Afterwards, the adjusted entry means were calculated using the emmeans package (Searle et al., 1980). This normalization method was designated in the following as EMM normalization. The GP performance was evaluated using, only EMM normalization, only TMM normalization, both normalization methods together (EMM first, TMM first), and with un-normalized read counts.

### Phenotypic datasets

In this study eight phenotypic traits were considered (Tab. 1). All phenotypic information was collected in field experiments using an augmented row-column design in which the RIL were planted with a single replicate. The parental inbreds were used as checks with multiple replicates. The awn and spike length data were collected between 2019 and 2021 in five different environments. Flowering time and plant height were part of field experiments from 2017 to 2019 in seven environments (Cosenza et al., 2024). Grain length, grain width, grain area, and thousand grain weight were measured between 2017 and 2019 in four different environments (Shrestha et al., 2022). The adjusted entry means for the phenotypic values were calculated using the following mixed linear model: $y_{ijk} = \mu + G_i + E_j + (G \times E)_{ij} + \varepsilon_{ijk}$, with the genotype effect ($G_i$, fixed), environment effect ($E_j$, random), and the genotype-environment interaction effect ($(G \times E)_{ij}$, random), as well as an error term ($\varepsilon_{ijk}$). The heritability for all phenotypic traits was calculated as $H^2 = \sigma_G^2/(\sigma_G^2 + \bar{\nu}/2)$, where $\bar{\nu}$ was the mean variance of difference between two adjusted entry means (Piepho and Möhring, 2007).

### Genomic prediction

We used all eleven datasets described above (Tab. 2) to predict the adjusted entry means for all eight traits. The values of all expression and sequence variant datasets were converted into z-scores ($\mu = 0$, $\sigma = 1$) for error normalization. Afterwards, additive relationship matrices were calculated for each of the datasets: $G = \frac{W^* {W^*}^T}{m}$, where $W^*$ was the z-score matrix of the feature measurements $W$, ${W^*}^T$ was the transposed z-score matrix and $m$ the number of features per dataset. We used the following genomic best linear unbiased prediction (GBLUP) model (VanRaden, 2008): $y = \mu + Zu + \varepsilon$, where $y$ was the vector of adjusted entry means of the examined trait, $\mu$ the general mean, $Z$ the incidence matrix of genotypic effects, and $u$ the vector of genotypic effects that are assumed to be normally distributed with $u \sim N(0, G\sigma_u^2)$, in which $G$ denotes the relationship matrix between inbreds and $\sigma_u^2$ the genetic variance. In addition, $\varepsilon$ is the vector of residuals following a normal distribution $\varepsilon \sim N(0, I\sigma_e^2)$. This approach was used across all three segregating populations. Multiple predictor datasets were included in the model by calculating the weighted average between the relationship matrices $G$ (Wu et al., 2022). The weight of each matrix was equal, if not otherwise stated. The training and testing population was simulated using 5-fold cross-validation and 50 repetitions.

### Genomic prediction: Intra and inter-population analyzes

In addition to the prediction across the three RIL populations, various intra- and inter-population analyses were performed. For the intra- and inter-population genomic prediction, fixed size randomized samples were used as training and testing set with 200 repetitions. The relationship matrices and GP model were identical to the above described 5-fold cross-validation method. We used multiple subsets from a single RIL population with a fixed number of individuals as training and testing set to evaluate the intra-population prediction ability of our data.

We created a training and testing set from the same RIL population. The number of individuals for each set was the same for all RIL populations. The inter-population prediction ability was tested by using one of the three RIL populations as training set and a second as testing set. The number of individuals included in both sets was kept constant. Intra- and inter-population results were compared to the GP performance using cross-population training and testing sets.

### Genomic prediction: Reduced sequencing depth

To evaluate the impact of reduced sequencing depth on GP performance, we tested 13 sequencing depth subsets. The number of reads per sequencing depth subset ranged from 10K to 7M. The reads to include were randomly selected from all uniquely mapped reads of the alignment data. Samples which did have less uniquely mapped reads than required were excluded. To ensure comparability, the same samples were included across all datasets for which GP was performed. All other steps of the data processing workflow were not changed. We tested the impact on RNA-Seq sequencing variant datasets for three different filtering methods creating the reduced sequencing depth equivalents of $SNP^{Total}_{RNAseq}$, $SNP^{Stn.}_{RNAseq}$, and $SNP^{QC}_{RNAseq}$ for each of the 13 sequencing depths. Similarly, the expression datasets were evaluated including all transcripts ($GE^{Total}_{RNAseq}$), filtered transcripts ($GE^{Filter.}_{RNAseq}$), and DEG ($GE^{DEG}_{RNAseq}$).

## RESULTS

### Characterization of gene expression data and their GP performance

RNA-Seq data was used to characterize the expression pattern of 237 RIL from the HvDRR13, HvDRR27, and HvDRR28 population and their parental inbreds Spratt-Archer, HOR8160, and Unumli-Arpa (Fig. 1). The number of detected transcripts per sample ranged from 22.1K to 30.9K (mean 28K) and did not show significant differences (p $>$ 0.05) between the genetic material groups (Fig. 2A). The different segregating populations were clearly separated in the expression-based PCA (Fig. 2 B). The RIL within each population were arranged according to their phenotypic values (Fig. S2).

The total gene expression dataset ($GE^{Total}_{RNAseq}$) which included 42.6K transcripts was divided into two subsets: (1) transcripts with an average cpm $>$ 0.39 ($GE^{Filter.}_{RNAseq}$; 37.7K transcripts) and (2) a subset only including DEG ($GE^{DEG}_{RNAseq}$; 7.3K transcripts)(Fig. 2C). We determined the cpm threshold for the $GE^{Filter.}_{RNAseq}$ dataset based on the maximum number of significant DEG detected (Fig. S3). This resulted in a cpm threshold lower than the standard edgeR threshold of about 1.7. The standard filtering approach of edgeR resulted in $<$ 6K DEG.

We measured gene expression per transcript as well as per gene and detected a slight but significant increase (p = 0.009) in PA for the former in $GE^{Total}_{RNAseq}$ (Fig. S4A). We also compared different normalization methods. For the un-normalized cpm and the TMM normalized cpm, the GP performance did not differ significantly (p $>$ 0.05), while the EMM normalized GP results were significantly lower ($p = 0.003$) compared to the PA of the un-normalized results (Fig. S4B-C).

The PA of the $GE_{RNAseq}^{Total}$ set was with an average 0.68 across all traits, the lowest of all gene expression subsets. The GP performance of $GE_{RNAseq}^{Filter.}$ was slightly higher (0.69) but both were outperformed by $GE_{RNAseq}^{DEG}$ which had an average prediction ability of 0.73 (Fig. 2D). The PA differed among the traits. Flowering time was in all subsets the most difficult to predict resulting in an average prediction ability between 0.59 and 0.67. Ear length was the trait most accurately predicted with an average prediction ability between 0.78 and 0.82.

### Prediction ability of sequence variant datasets

In addition to the expression data, sequence variant calling was performed for the RNA-Seq dataset comprising 148K variants after cleaning and imputation ($SNP_{RNAseq}^{Total}$). The SNP data was further subset based on variant function annotation, creating a set that only included variants in regulatory regions of genes ($SNP_{RNAseq}^{Reg.}$) and a set that only included missense variants in gene coding sequences ($SNP_{RNAseq}^{CDS}$). The latter led to an increase in PA of 0.013 compared to $SNP_{RNAseq}^{Total}$ (Fig. 3). A comparable increase in PA was achieved by filtering the RNA-Seq variants using quality criteria.

In this study, two quality-filtered subsets were evaluated. First, our own empirical quality filtering subset designated in the following as $SNP_{RNAseq}^{QC}$ and second, the subset $SNP_{RNAseq}^{Stn.}$ which is based on previously published filtering criteria (Atanda et al., 2022; Wen et al., 2018). We used the following quality criteria for the empirical quality filtering workflow: read depth (DP), minor allele frequency (MAF), missing data (NS), and quality score (QUAL). More than 20 quality filtering subsets of $SNP_{RNAseq}^{Total}$, ranging from no filtering to maximum filtering strength, were created and their GP performance was evaluated (Fig. S5). For each of the quality filtering criteria, the best performing $SNP^{Total}_{RNAseq}$ subset was selected and combined with one or more of the remaining subsets (Fig. 4A). Most combinations showed a slight increase in PA and the best performing combination (designated in the following as $SNP^{QC}_{RNAseq}$) was 0.002 higher than the subset of best performing single criteria of the combination (not significant: p > 0.05). $SNP^{QC}_{RNAseq}$ included 42.5K variants, utilized MAF and QUAL as quality filter criteria, and performed with a 0.015 PA increase considerably better than $SNP^{Total}_{RNAseq}$. A slightly lower uplift was achieved by $SNP^{Stn.}_{RNAseq}$. However, when comparing the GP results of $SNP^{Stn.}_{RNAseq}$ and $SNP^{QC}_{RNAseq}$ for each of the traits, $SNP^{QC}_{RNAseq}$ performed significantly better ($p \leq 0.025$) for half of them (Fig. 4B). A PCA was performed using the $SNP^{QC}_{RNAseq}$ subset and a separation between the three parental inbreds and RIL populations was observed as expected (Fig. S6A). Each of the eight traits showed clear differences among the parental inbreds that caused phenotypic variance in the corresponding segregating populations (Fig. S6B-I).

Lastly, WGS data from the parental inbreds were used to create an optimized WGS imputed RNA-Seq consensus variant dataset ($SNP^{Total}_{WGS}$) achieving a PA of 0.776. This constitutes a 0.033 increase compared to $SNP^{QC}_{RNAseq}$ and 0.009 compared to the SNP array dataset. $SNP^{Total}_{WGS}$ showed the highest PA averaged across all traits. Based on $SNP^{Total}_{WGS}$, two function-based subsets ($SNP^{Reg.}_{WGS}$ and $SNP^{CDS}_{WGS}$) were created, but none of them had a positive impact on the GP results.

### GP performance of combined datasets

We selected the best performing datasets from these four main groups: gene expression ($GE^{DEG}_{RNAseq}$), RNA-Seq SNP ($SNP^{QC}_{RNAseq}$), RNA-Seq/WGS consensus SNP ($SNP^{Total}_{WGS}$), and array SNP ($SNP^{Total}_{Array}$). We tested the combined GP performance by merging two or more of these datasets (Fig. 5). For that, the additive relationship matrices calculated from each of the individual datasets were averaged, and the resulting matrix was used for GP. The combination of two datasets resulted in an average increase in PA of 0.01. Three of the six combined matrices were able to outperform the best dataset included in the combination (Fig. 5A). The average performance of all triple dataset combinations (0.77) was increased by 0.02 compared to the single datasets and by 0.01 compared to the double dataset combinations. Combining all four datasets resulted in an average PA of 0.78, which is 0.02 higher compared to the average single dataset GP and slightly better than the triple dataset combination average. However, none of the combinations showed a significantly higher PA (p > 0.05) than the best individual dataset included in the combination.

Examining each trait separately revealed a significant (p < 0.01) increase in GP performance for at least one of the traits in multiple combined datasets (Fig. 5B). The best performing combination consisted of $SNP_{RNAseq}^{QC}$ and $SNP_{Array}^{Total}$, as it significantly increased PA for three traits ($p \leq 0.016$) and only decreased significantly (p < 0.001) once. Combining $SNP_{WGS}^{Total}$ and $GE_{RNAseq}^{DEG}$ with equal weight resulted in a significant decrease in prediction ability for all eight traits. We also tested for significant differences compared to $SNP_{Array}^{Total}$ (Tab. S1). $SNP_{WGS}^{Total}$ significantly outperform $SNP_{Array}^{Total}$ in five of eight traits. Combining $SNP_{WGS}^{Total}$, $SNP_{RNAseq}^{QC}$ and $GE_{RNAseq}^{DEG}$ significantly increased half of the traits ($p \leq 0.025$), which was the best-performing combination without including $SNP_{Array}^{Total}$. We further evaluated the combination including $GE_{RNAseq}^{DEG}$ and $SNP_{WGS}^{Total}$ by testing a range of weighted combinations (Fig. 5C). We started with only the $GE_{RNAseq}^{DEG}$ dataset and gradually increased the percentage of weight contribution $SNP_{WGS}^{Total}$ until only the $SNP_{WGS}^{Total}$ data remained.

The results of the trait flowering time showed a significant increase (p = 0.007) in GP performance for an unbalanced combination with a 90% weight contribution by $SNP^{Total}_{WGS}$. For all other traits, the combinations resulted in lower PA.

We quantified the differences of the same four datasets by calculating Pearson's correlation coefficient of the additive relationship matrices (Fig. S7). The correlation between $SNP^{QC}_{RNAseq}$ and $SNP^{Total}_{WGS}$ was the highest with a correlation coefficient of 0.98. The correlation of $SNP^{Total}_{Array}$ with $SNP^{Total}_{WGS}$ ($r = 0.91$) and $SNP^{QC}_{RNAseq}$ ($r = 0.89$) showed slightly lower level of similarity. The gene expression matrix ($GE^{DEG}_{RNAseq}$) was more dissimilar compared to the remaining datasets ($r = 0.59 - 0.61$). We split the additive relationship matrices into segments based on the populations to calculate the correlation for all populations and inter-population combinations separately. We focused on the correlation between $SNP^{Total}_{Array}$ and the remaining three datasets (Fig. S8) and showed for all three matrix combinations that the correlation coefficient was lower for the intra-population covariances than for the inter-population covariances. Additionally, large differences in similarity were shown for intra-population covariances.

### Comparison of intra- vs. inter-population GP

To compare the GP performance using population-specific combinations of training (TS) and validation sets (VS), we had to adjust the validation procedure to account for the variable population sizes. Therefore, we switched from a 5-fold cross-validation scheme to a random subset validation with fixed training and testing set sizes. We established a baseline by performing cross-population GP using samples of multiple populations in TS and VS (Fig. 6A). The prediction ability with a low TS size (20) varied between 0.47 ($GE^{DEG}_{RNAseq}$) and 0.58 ($SNP^{Total}_{Array}$). These results could be improved by 0.25 for $GE^{DEG}_{RNAseq}$ and by 0.18 for $SNP^{Total}_{Array}$ when increasing TS size to 170.

We then separated the populations and tested intra-population GP performance (TS = VS population) and inter-population GP performance (TS $\neq$ VS population) with a fixed TS size of 50 (Fig. 6B). For most datasets and comparisons, the intra-population GP results were slightly lower than cross-population results, with only $SNP^{QC}_{RNAseq}$ and $SNP^{Total}_{WGS}$ showing slightly higher performance (0.02) for HvDRR28. The prediction abilities of the inter-population GP results were lower compared to the intra-population GP results. GP performance depended on the combination of populations tested and resulted in prediction abilities ranging from 0 to 0.29. The worst performing combination between HvDRR28 (TS) and HvDRR13 (VS) was best predicted by $GE^{DEG}_{RNAseq}$.

Although the overall ranking between the datasets did not change between intra- and inter-population GP (Fig. S9), the relative differences between them varied (Fig. 6C). $SNP^{Total}_{WGS}$ performed 5% better than $SNP^{QC}_{RNAseq}$ in intra-population GP and the difference increased to 25% in inter-population GP. The difference in PA between $SNP^{Total}_{WGS}$ and $SNP^{Total}_{Array}$ increased from $< 1\%$ in intra-population GP to almost 3% better performance of $SNP^{Total}_{WGS}$ in inter-population GP. The difference in performance between $SNP^{QC}_{RNAseq}$ and $GE^{DEG}_{RNAseq}$ was largest in intra-population GP (13%) but decreased to 3% in inter-population GP aligning the performance of both datasets.

### Effect of sequencing depth reduction on GP performance

We tested the GP performance of 13 sub-sampled datasets with reduced sequencing depths from 7M to 10K reads using RNA-Seq SNP and gene expression data including 155 samples with sufficient reads (Fig. S10) and from 3M to 10K reads including 229 samples (Fig. 7). The GP performance of the three RNA-Seq SNP datasets was significantly ($p < 0.001$) affected by a reduction in sequencing depth (Fig. 7A). For $SNP^{Total}_{RNAseq}$, the prediction ability decreased only insignificantly ($p > 0.05$) for all subsets that started with more than 400K uniquely mapped reads (200K for $SNP^{Stn.}_{RNAseq}$ and $SNP^{QC}_{RNAseq}$). Reduction in sequencing depth below 100K reads impacted the GP performance considerably more. $SNP^{Total}_{RNAseq}$ showed higher prediction ability values than $SNP^{Stn.}_{RNAseq}$ below 50K variants, while the quality filtering dataset $SNP^{QC}_{RNAseq}$ increased the performance in all subsets. For the three gene expression datasets, performance started to decline earlier, with only the 2M and 3M subsets showing no significant differences between each other ($p > 0.05$) (Fig. 7B). $GE^{DEG}_{RNAseq}$ performed worst in the 10K subset and only exceeded the prediction ability values of the other two datasets at sequencing depths over 100K.

## DISCUSSION

### Filtering of RNA-Seq datasets to unravel the full GP potential

We started with independently evaluating the GP performance of sequence variants and gene expression datasets derived from RNA-Seq data in a cross-population scenario. For most applications, the accuracy of RNA-Seq gene expression data is increased by the inclusion of replicates to counter act the technical errors introduced by experimental factors (Schurch et al., 2016). However, we expect that RNA-Seq will only find its way into routine plant breeding programs, if it can be applied without replications and as such it was examined in our study. Therefore, we normalized the gene expression values per transcript based on a linear model including a block effect to adjust the read counts (EMM) (Fig. S4B). While in 88% of all transcripts in $GE^{DEG}_{RNAseq}$ the block effect was significant (p < 0.05), adjusted gene expression values negatively affected GP performance and led to a significant PA decrease ($p < 0.001$). Further research is necessary to learn whether a block normalization can be helpful in normalizing gene expression data in a controlled environmental setting similar to this study.

In addition to the EMM normalization, we tried to account for differences in transcriptome composition or sequencing lane effects by applying the per-sample normalization TMM implemented in edgeR (Robinson and Oshlack, 2010; Dillies et al., 2013), but it did not significantly (p > 0.05) change the prediction ability of $GE^{DEG}_{RNAseq}$ (Fig. S4C). We assume that this is because all examined genotypes were partially related, limiting differences in transcriptome composition. Furthermore, the library pool was evenly distributed across all lanes.

Although normalization did not lead to an uplift in GP performance, we were able to increase PA by filtering for DEG. The resulting dataset $GE^{DEG}_{RNAseq}$ was the best performing gene expression dataset across all traits with an average prediction ability of 0.73 (Fig. 2). Filtering also increased GP performance in the sequence variant datasets extracted from the RNA-Seq data (Fig. 3). In the end, the prediction based on sequence variation exceeded the prediction based on gene expression when averaged across all traits. This finding is in agreement with previous results (Azodi et al., 2020). This indicates a limited prediction potential of gene expression data which could have multiple reasons. Similarly to Azodi et al. (2020), we were using only seedling material for GP. Therefore, most spatiotemporal specific DEG were not captured in our data (Klepikova et al., 2016), which potentially limits GP performance. Conversely, creating trait-specific transcriptome data by focusing on the most relevant time point, developmental stage, and tissue could increase the ability to predict specific traits.This, however, is unlikely to be realistic in the context of commercial plant breeding programs. The overall number of transcripts detected was limited by the sequencing depth (Conesa et al., 2016). However, the sequencing depth in our study was chosen so that the related costs are comparable to that of genotyping using a SNP array. Ultimately, the prediction ability potential of transcriptome datasets can only be fully exploited by grouping and weighting individual genes, using prior knowledge of the trait and associated gene networks. Similar methods exist but have never been applied in this context (Zarringhalam et al., 2018). We are convinced that future research can further increase the GP performance of transcriptome data, while the costs of creating such datasets will remain comparable to the costs of an SNP array.

We evaluated the impact of function filtering of the unfiltered RNA-Seq based variant data ($SNP^{Total}_{RNAseq}$) based on two criteria: SNP in the 3'UTR and 5'UTR and nonsynonymous SNP in the CDS. In our study, both function filtering criteria achieved comparable GP performance while relying only on a fraction of the total variants, which also was previously observed (Li et al., 2022; Cappetta et al., 2021; Tan et al., 2017). While the 3'UTR and 5'UTR are known to be regulatory regions in which SNP can be associated with gene expression variation (Dossa et al., 2021; Zhang et al., 2019), in our study filtering missense SNP in the CDS resulted in a greater uplift of GP performance.

Quality filtering using the four quality criteria read depth, minor allele frequency, missing rate, and quality score were able to increase prediction ability compared to $SNP^{Total}_{RNAseq}$. The combination of multiple quality filter criteria further increased the GP results and led to the best-performing RNA-Seq sequence variant dataset $SNP^{QC}_{RNAseq}$ (Fig. 4). Although our empirical quality filtering workflow did not result in a much larger average prediction ability compared to the commonly used filter settings (Atanda et al., 2022; Wen et al., 2018), for half of the traits evaluated in this study, PA was significantly increased ($p \leq 0.025$) by empirical quality filtering. We leveraged the information of the homozygosity of the RILs, the population structure, and the parental information to strictly clean and correct the sequence variant data, which reduced the impact of quality filtering on the GP performance. However, we observed that without strict cleaning using the before mentioned prior knowledge, the impact of quality filtering was much greater (Fig. S11). We applied the empirical quality filtering workflow to $SNP^{Raw}_{RNAseq}$, which resulted in a prediction ability of 0.75 which is comparable to that of $SNP^{QC}_{RNAseq}$. This observation suggests that our empirical quality filtering workflow is therefore also able to provide good prediction abilities in situations with limited prior knowledge and, thus, might be relevant for other studies.

The last approach to improve the prediction ability that we examined was the exploitation of SNPs from parental inbred lines. In our study, the consensus dataset from the RNA-Seq and WGS variants ($SNP^{Total}_{WGS}$) was the overall best performing dataset for GP. $SNP^{Total}_{WGS}$ outperformed $SNP^{Total}_{Array}$ for five out of eight traits significantly ($p < 0.05$). Adding WGS data to improve GP performance was previously only examined in combination with SNP array data (Weber et al., 2024; Brøndum et al., 2015). Weber et al. (2024) showed insignificant improvements to the prediction accuracy and concluded that the WGS data were unable to add important information due to the relatedness of the sample groups and linkage disequilibrium between many WGS markers. In contrast, in our study the advantage of including WGS data is apparent and illustrates that for some predictor types, for instance, RNA-Seq and potentially also GBS datasets, the benefit from WGS data is more significant than for the already highly curated markers included in a SNP Array.

### Cost optimization of GP using RNA-Seq

In addition to the filtering steps discussed above to improve the prediction ability, the selection of the optimal TS size is also important. In our study, increasing the TS size from 20 to 80 led to a drastically increased prediction ability (Fig. 6A). Increasing the TS to 170 only slightly increased the prediction ability further. The diminishing returns of TS size depends on the population structure and has been studied extensively before and therefore are not discussed further (Zhu et al., 2022; Bustos-Korts et al., 2016; Isidro et al., 2015; Rincent et al., 2012; Lorenz et al., 2012).

One further aspect to optimize the balance between prediction ability and cost is the sequencing depth. Our resampling simulations illustrated that the sequencing depth of RNA-Seq can be significantly reduced without sacrificing GP performance (Fig. 7). For $SNP^{QC}_{RNAseq}$, the reduction in GP performance was only marginal until the number of reads fell below 200K. This finding indicated that many sequence variants do not contain additional information, as they are part of the same haplotype block, and therefore reducing the number of reads and therewith sequence variants does not necessarily lead to a decreased trait prediction.

However, when reducing the sequencing depth further, we observed for the datasets that underwent a strong quality filtering a considerably reduction in PA. The $SNP^{Stn.}_{RNAseq}$ filtering resulted in $< 100$ sequence variants in the 10K read depth subset compared to the 6.9K marker in $SNP^{Total}_{RNAseq}$. Therefore, GP performance decreased as a consequence due to an insufficient number of polymorphic variants, which in turn led to an imprecise estimation of genetic relatedness. $SNP^{Stn.}_{RNAseq}$ and $SNP^{Total}_{RNAseq}$ were outperformed by $SNP^{QC}_{RNAseq}$ with 1.5K sequence variants at the same sequencing depth. These results illustrate the balance between the number of variants needed to achieve the necessary LD between variants and trait-coding polymorphisms and the selection of informative variants.

In comparison to the above outlined influence of a reduced sequencing depth on the prediction ability of sequence variant datasets, we observed a reduction of the prediction ability already at higher number of reads (around 3M) for the gene expression datasets. Even more pronounced was that the reduced number of reads led to a reduction in statistical power to detect DEG. For example, 400K reads were able to detect 76% of the transcripts of the total dataset, but only 22% of the DEG.

This ultimately resulted in less than 100 DEG in sequencing depth subsets below 20K, which led to dramatically reduced prediction abilities.

With reduced sequencing depth, the sequencing is most likely no longer the most expensive step of the project, and the remaining cost saving potential can be realized by adjusting the library preparation workflow. In this project, we used a thoroughly evaluated miniaturization workflow that reduced the costs of the library preparation by a factor of four (Arlt et al., 2023). At reduced sequencing depth, as discussed above, such approaches to reduce library preparation costs are even more crucial compared to our study.

### Combining multiple datasets maximized GP performance

Besides the above described independent evaluations of the sequence variants and gene expression datasets, we also examined various scenarios on combining them. In detail, the overall best performing dataset $SNP^{Total}_{WGS}$, the gene expression dataset $GE^{DEG}_{RNAseq}$, the RNA-Seq sequence variance dataset $SNP^{QC}_{RNAseq}$, and the 50k SNP array dataset $SNP^{Total}_{Array}$ were selected to be examined for their joint prediction ability. We observed a clear positive trend in PA when combining these datasets (Fig. 5). This was expected because their different information content complement each other.

However, not all combinations of individual datasets outperformed the best single predictor. The GP performance of all combinations was significantly decreased ($< 0.05$) for at least one trait. This highly trait-specific GP performance was in accordance with previous reports (Zhu et al., 2022; Michel et al., 2021) and can be explained by their differences in heritability and the number of small- and large-effect loci contributing to the trait. The high similarities between the sequence variant datasets $SNP^{QC}_{RNAseq}$, $SNP^{Total}_{WGS}$, and $SNP^{Total}_{Array}$ (Fig. S7) could explain the limited potential of their combinations to increase GP performance. The correlation coefficient indicated more differences between $GE^{DEG}_{RNAseq}$ and $SNP^{Total}_{Array}$ or $SNP^{QC}_{RNAseq}$, but combining them resulted only for two traits in significantly increased PA ($p \leq 0.014$). No clear increase in GP performance was observed by adding gene expression as an additional information layer to sequence variants, which was consistent with previous studies (Azodi et al., 2020; Guo et al., 2016).

In the first chapter of the discussion, we listed potential reasons for the limited PA potential of gene expression data and how to improve them. It is not clear whether such an improved transcriptome dateset would better complement or replace sequence variant data for the purpose of genomic prediction analyses. We also see a limited potential for datasets like ours to be further improved. For example, using logarithmic scaling of read counts increased the correlation coefficient between $GE^{DEG}_{RNAseq}$ and the remaining datasets by 2-3% and slightly increased GP performance. This indicates that GP similar to differential expression analysis or co-expression analysis profits from the increased homoscedasticity of log-scaled expression values (Johnson and Krishnan, 2022; Love et al., 2014). Log-scaling could be especially beneficial when gene expression data are later combined with SNP data. Further research is needed to test this hypothesis.

### Genetic diversity vs. relatedness: cross-, inter-, and intra-population prediction

Our multi-parent recombinant inbred line population allowed us to test multiple GP population designs and evaluate their performance using different input datasets (Fig. 6). Combining multiple populations in the TS (cross-population) increased the GP

performance in our study. This finding was in accordance with the results of Berro et al. (2019), but contrary to those of Lorenz et al. (2012) and illustrates that the ratio between population structure and diversity dictates whether the creation of a cross-population TS is beneficial.

The relative difference in PA between $SNP_{WGS}^{Total}$ and the remaining datasets was higher for the inter-population GP vs. the intra-population GP (Fig. 6C). This could be explained by the increased marker count of $SNP_{WGS}^{Total}$, which could have led to a higher linkage between marker and QTL, which was shown to be more relevant when the genetic distance between TS and VS is increased (De Roos et al., 2009). Also noticeable, $GE_{RNAseq}^{DEG}$ and $SNP_{RNAseq}^{QC}$ performed similarly in inter-population GP, while in intra-population GP $SNP_{RNAseq}^{QC}$ was clearly superior. This shift was mainly caused by a single inter-population combination that could be predicted by $GE_{RNAseq}^{DEG}$ but not by $SNP_{RNAseq}^{QC}$.

### SUMMARY

In this study, we evaluated various approaches to optimize the prediction potential of RNA-Seq datasets. Our results indicated that the RNA-Seq sequence data in combination with parental sequence data out-performed the SNP array data in five out of eight traits, showing a higher prediction ability overall. The outstanding performance of the consensus dataset combining WGS genomic variant information of the parental lines with the RNA-Seq sequence variant data is highly relevant. More and more diverse and for breeding relevant barley genotypes are sequenced, the amount of publicly accessible data will increase, allowing for comparable data combinations in the future to increase the GP potential in barley. The same is also true for other crop species.

When relying solely on RNA-Seq data, we showed that focusing on differentially expressed genes noticeably increased the prediction ability of the gene expression dataset but did not reach the prediction ability level of the sequence variant datasets. The GP performance of the RNA-Seq sequence variant data was substantially increased by strict data cleaning and quality filtering, but it only exceeded the prediction performance of the SNP array for one trait. Combining predictor datasets does not significantly ($p > 0.05$) increase PA averaged across all traits, but was able to significancy increase individual traits in half of the combinations tested. Therefore, the expected benefit of combing the RNA-Seq sequence variant and gene expression data did not materialize for our genetic material.

In this study, we showed that low-cost and high-throughput RNA-Seq data can achieve comparable or better GP performance than traditional SNP array datasets.

As the RNA-Seq data are more flexible, not relying on a pre-selected set of genomic variance, and allow for further decreased costs by adjusting the read depth and library preparation protocols, we see high application potential for RNA-Seq in plant breeding programs.

## STATEMENTS & DECLARATIONS

### Funding

This research is funded by the Deutsche Forschungsgemeinschaft (DFG, German Research Foundation) under Germany's Excellence Strategy (EXC 2048/1, Project ID:390686111).

### Competing Interests

Benjamin Stich is a member of the editorial board of *Theoretical and Applied Genetics*. Furthermore, the authors have no competing interests to declare that are relevant to the content of this article.

### Author contributions

The study was conceptualized and designed by C.A and B.S.; The RIL population was designed by B.S and J.L; The genetic material was propagated and maintained by D.I and B.S; The data were analyzed and interpreted by C.A and B.S; The manuscript was written by C.A. and edited by C.A and B.S; All authors read and approved the final manuscript.

### Data availability

The raw read RNA-Seq dataset analyzed in this study is available in the NCBI Sequence Read Archive (SRA), BioProject ID: PRJNA1088431, URL:

https://www.ncbi.nlm.nih.gov/bioproject/1088431

https://www.ncbi.nlm.nih.gov/sra/PRJNA1088431

**Table 1.** Overview of all studied phenotypic traits. Number of environments in which the trait was assessed, broad sense heritability ($H^2$), minimum (Min.), maximum (Max.), median, mean, and standard deviation (SD) of adjusted entry means of all traits included in this study.

| Traits | Environments | $H^2$ | Min. | Max. | Median | Mean | SD |
|---|---|---|---|---|---|---|---|
| Ear/spike length [cm] | 5 | 0.88 | 6.04 | 13.64 | 8.96 | 8.92 | 1.28 |
| Awn length [cm] | 5 | 0.74 | 10.07 | 17.60 | 13.45 | 13.52 | 1.39 |
| Flowering time [d] | 7 | 0.86 | 55.73 | 87.74 | 70.36 | 69.62 | 6.10 |
| Plant height [cm] | 5 | 0.76 | 38.90 | 99.28 | 69.60 | 69.67 | 9.34 |
| Grain length [mm] | 4 | 0.84 | 7.35 | 13.92 | 10.69 | 10.84 | 1.02 |
| Grain width [mm] | 4 | 0.85 | 2.80 | 3.76 | 3.41 | 3.40 | 0.18 |
| Grain area [$mm^2$] | 4 | 0.89 | 16.48 | 30.83 | 25.59 | 25.28 | 2.66 |
| Thousand grain weight [g] | 4 | 0.84 | 25.10 | 55.09 | 37.76 | 37.96 | 5.73 |

**Table 2.** Overview of all predictor datasets. Name, abbreviation, origin, and number of features (before filtering) for all genomic and transcriptomic datasets included in this study.

| Name | Abbreviation | Origin | No. of features |
|---|---|---|---|
| Total transcript expression | $GE_{RNAseq}^{Total}$ | RNA-Seq | 42.6K |
| Filtered transcript expression | $GE_{RNAseq}^{Filter.}$ | RNA-Seq | 37.7K |
| Differentially expressed transcript expression | $GE_{RNAseq}^{DEG}$ | RNA-Seq | 7.3K |
| Unfiltered transcriptome sequence variants | $SNP_{RNAseq}^{Total}$ | RNA-Seq | 147.5K |
| Functional transcriptome sequence regulatory variants | $SNP_{RNAseq}^{Reg.}$ | RNA-Seq | 81.7K |
| Functional transcriptome sequence non-synonymous variants | $SNP_{RNAseq}^{CDS}$ | RNA-Seq | 25.8K |
| Standard filtered transcriptome sequence variants | $SNP_{RNAseq}^{Stn.}$ | RNA-Seq | 52.2K |
| Quality filtered transcriptome sequence variants | $SNP_{RNAseq}^{QC}$ | RNA-Seq | 42.5K |
| RNA-Seq / WGS consensus sequence variants | $SNP_{WGS}^{Total}$ | Hybrid | 426.4K |
| RNA-Seq / WGS consensus sequence non-synonymous variants | $SNP_{WGS}^{CDS}$ | Hybrid | 52.4K |
| RNA-Seq / WGS consensus sequence regulatory variants | $SNP_{WGS}^{Reg.}$ | Hybrid | 245.6K |
| 50k SNP array data | $SNP_{Array}^{Total}$ | Array | 17.3K |

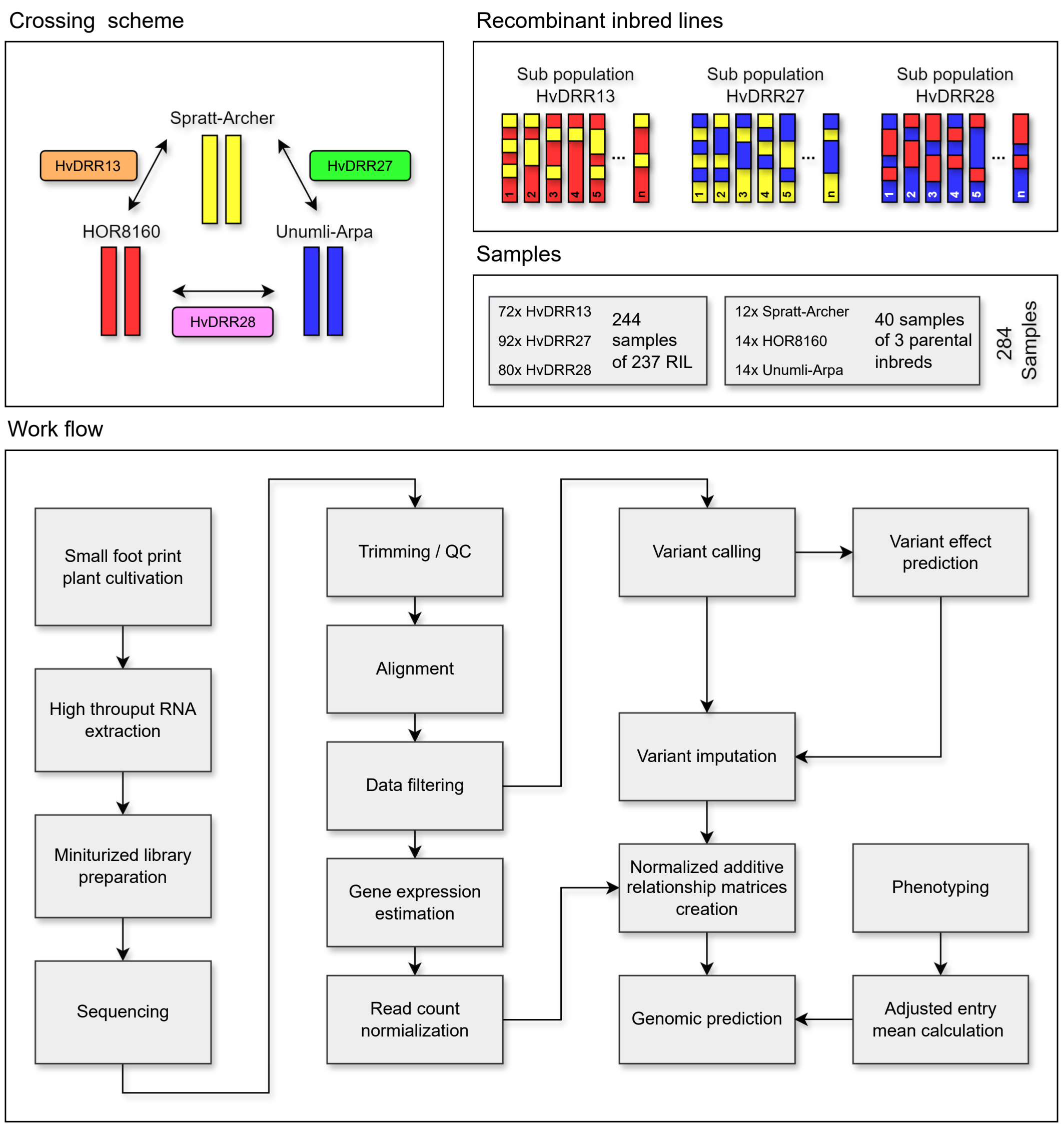

**Fig. 1.** Genetic material and workflow overview. The crossing scheme shows the three homozygous parental inbreds that were used to create the recombinant inbred line populations HvDRR13, HvDRR27, and HvDRR28 (F7/F8). The workflow is shown by connecting the major steps in consecutive order.

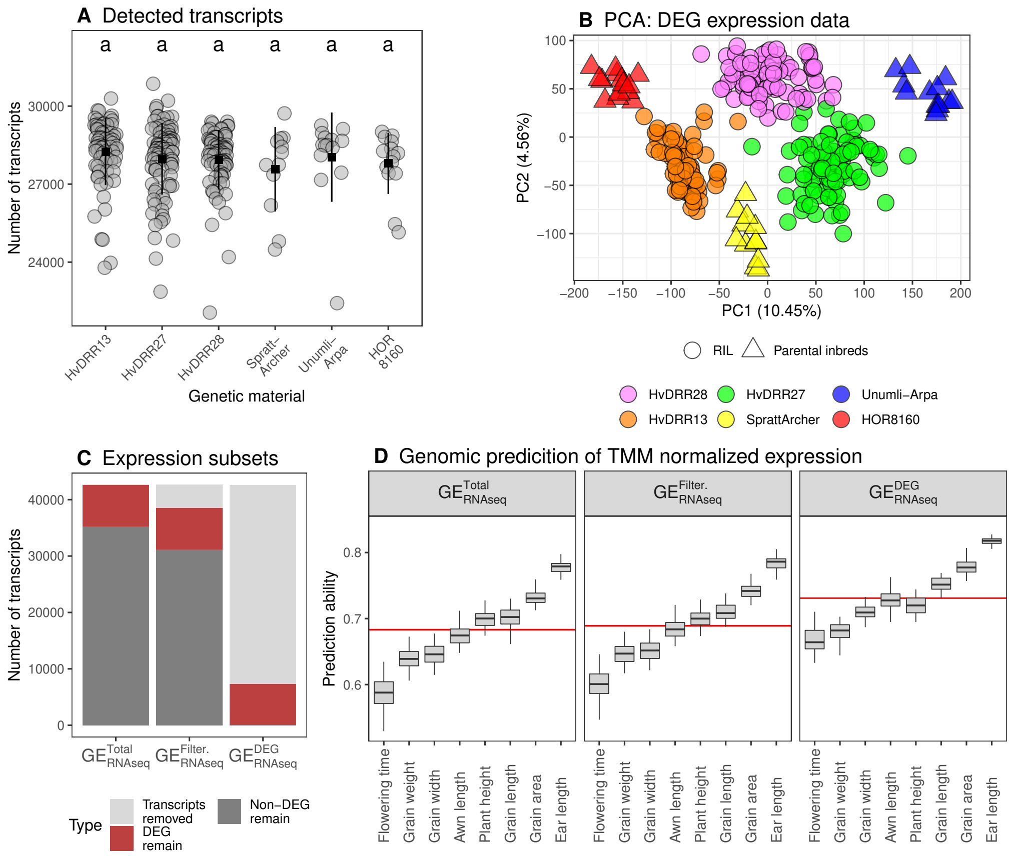

**Fig. 2.** Characteristics and genomic prediction (GP) performance of the gene expression datasets. (A) The number of detected transcripts are grouped by genetic material with mean (black square) and standard deviation (black line). Populations marked by the same letter are not significantly ($\alpha = 0.05$) different from each other. (B) Principle component analysis using the differentially expressed genes (DEG) subset ($GE_{RNAseq}^{DEG}$). PC 1 and PC 2 are the first and second principal component, respectively, and the number in parentheses refers to the proportion of variance explained by the principal components in percent. (C) Number of transcripts included in all data subsets used for genomic prediction (GP). (D) GP results are compared between all gene expression subsets and all traits included in this study. The red line shows the mean prediction ability per subset.

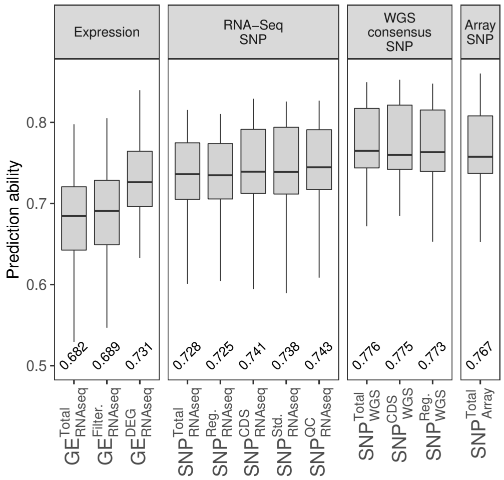

**Fig. 3.** Prediction ability of four dataset groups: Expression, RNA-Seq SNP, WGS consensus SNP, and SNP array across all the eight examined traits. For each dataset, the mean prediction ability value is shown as number in the bottom.

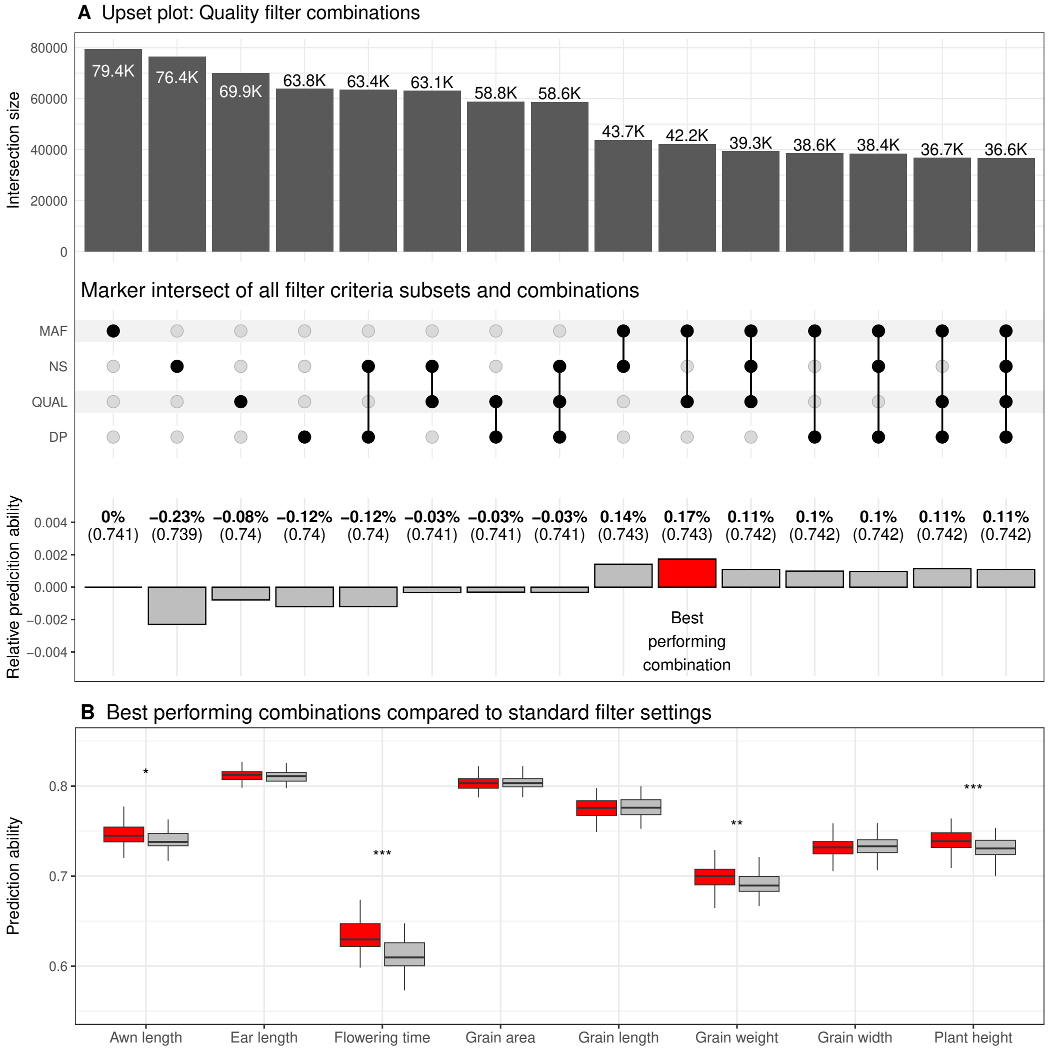

**Fig. 4.** (A) Combination of quality filtering subsets of genomic variants and their relative genomic prediction (GP) performance. For each of the combinations, the number of remaining variants (top) are shown after selecting the inclusive intersect between all included filtering subsets (middle). The four quality filtering criteria are minor allele frequency (MAF), variant quality score (QUAL), missing rate (NS), and read depth (DP). The subsets are the best performing subsets that were marked in Fig 4 with "T". The relative GP performance (bottom) shows the difference in prediction ability in percent between the combined subsets and the top performing single quality filtered subset (DP). The best performing subset is marked (red). (B) GP performance comparison between a standard quality filtering subset (white) (NA $<$ 20%, MAF $>$ 0.05) and the best performing combination (red). The GP results were averaged across all eight traits. Significant differences: p-value $<$ 0.01 = **, p-value $<$ 0.001 = ***.

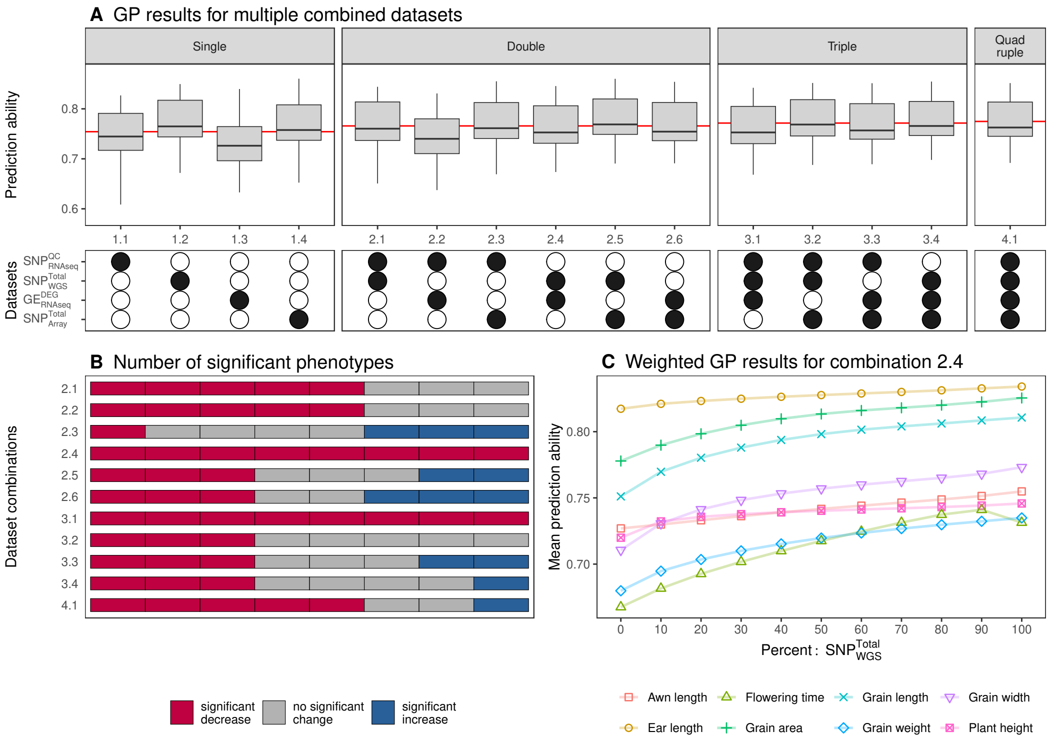

**Fig. 5.** Overview of genomic prediction abilities using multiple datasets. (A) The four different single datasets: $SNP^{QC}_{RNAseq}$, $SNP^{Total}_{WGS}$, $GE^{DEG}_{RNAseq}$, and $SNP^{Total}_{Array}$ were combined and prediction ability was calculated (top). The combinations are indicated as a combination of white (not included) and black (included) dots (bottom). The red line shows the mean prediction ability within each combination group. (B) The number of significantly (p $>$ 0.05) increased (blue), decreased (red), and not significantly changed (grey) phenotypic traits compared to the value of the single best dataset for each combination. (C) The mean prediction ability is plotted for the weighted combination of $SNP^{Total}_{WGS}$ and $GE^{DEG}_{RNAseq}$ (2.4). The weight is gradually changed from 100% $GE^{DEG}_{RNAseq}$ (left) to 100% $SNP^{Total}_{WGS}$ (right) with a step-size of 10%. The results are shown for each weighted combination and all traits.

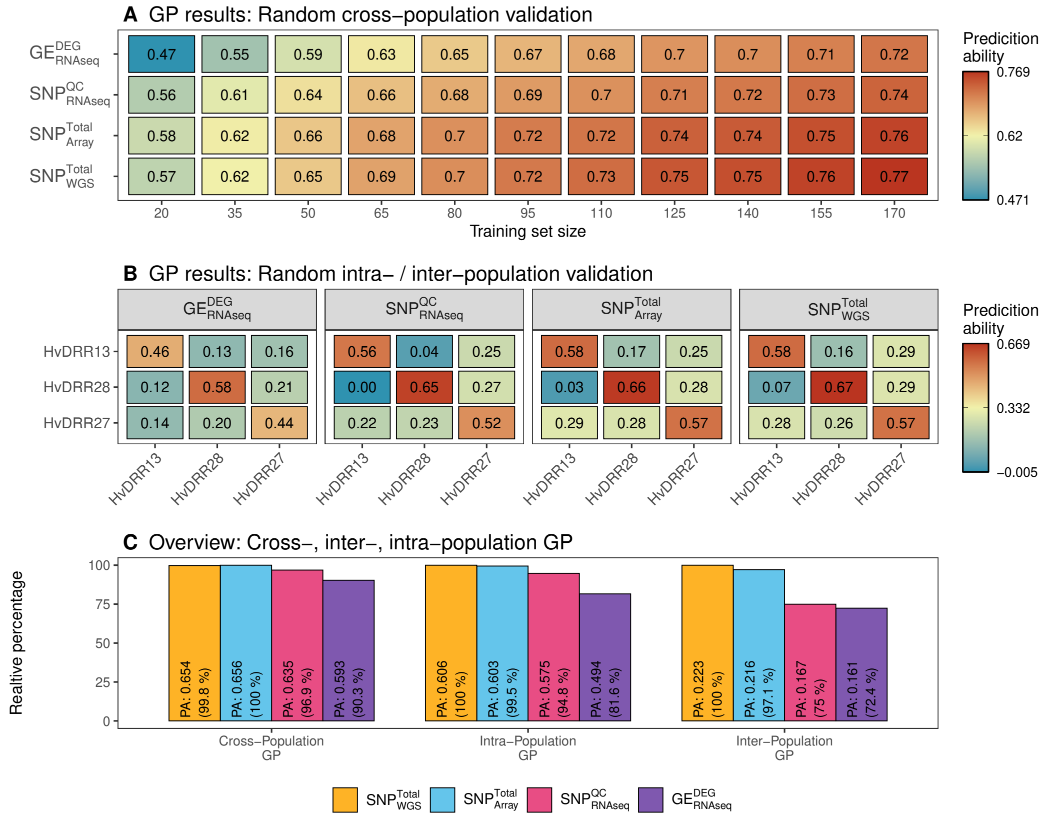

**Fig. 6.** (A) Genomic prediction (GP) results of the best performing datasets from each data origin group using randomly selected cross-population training sets (TS). The TS size was gradually increased from 20 to 170 individuals (step-size: 15). (B) GP results for intra-population (diagonal) or inter-population (off diagonal) comparisons. (C) Prediction ability (PA) deviation between the four main datasets for cross-, intra-, and inter-population genomic prediction. PA was averaged over all random validation runs and all intra- and inter-population pairwise comparisons. The training sets sizes was set to 50 for (B) and (C).

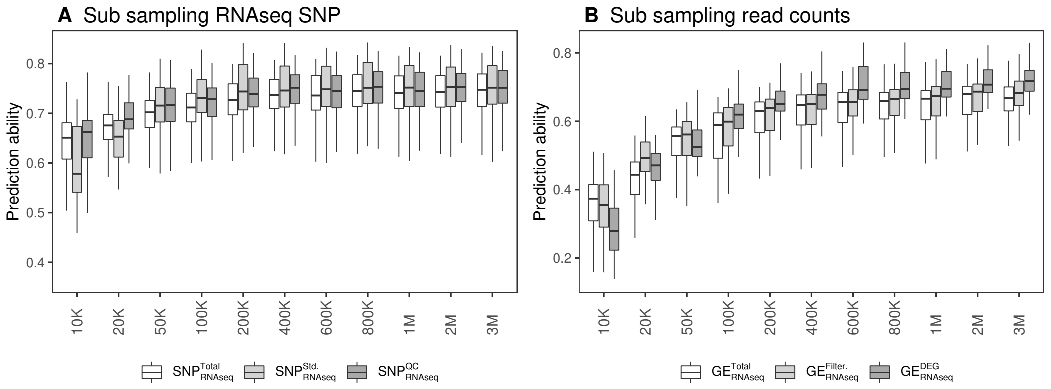

**Fig. 7.** Genomic prediction (GP) performance of artificially reduced sequencing depth subsets created by random read sub-sampling of (A) RNA-Seq genomic variant datasets ($SNP^{Total}_{RNAseq}$, $SNP^{QC}_{RNAseq}$) and (B) gene expression datasets ($GE^{Total}_{RNAseq}$, $GE^{Filter.}_{RNAseq}$, $GE^{DEG}_{RNAseq}$) including 229 of the 240 samples. The sub-sampling ranged in 11 steps between 10 thousand to 3 million reads.

**Table S1.** GP performance changes of all dataset combinations compared to $SNP_{Array}^{Total}$ (here: 1.4). All traits with significantly increased prediction abilities (Up), significantly decreased prediction abilities (Down), and not significantly changed prediction abilities (NC) were counted and named (EL: ear length, AL: awn length, PH: plant height, FT: flowering time, GL: grain length, GW: grain width, GA: grain area, TGW: grain weight). Dataset names are analogous to Fig. 5.

| Dataset | Down | Up | NC | Decreased traits | Increased traits |
|---|---|---|---|---|---|
| 1.1 | 7 | 1 | 0 | EL,PH,FT,GL,GW,GA,TGW | AL |
| 1.2 | 2 | 5 | 1 | GA,TGW | EL,AL,FT,GL,GW |
| 1.3 | 7 | 0 | 1 | AL,PH,FT,GL,GW,GA,TGW | |
| 1.4 | 0 | 0 | 8 | | |
| 2.1 | 2 | 4 | 2 | GA,TGW | EL,AL,FT,GL |
| 2.2 | 7 | 1 | 0 | EL,PH,FT,GL,GW,GA,TGW | AL |
| 2.3 | 1 | 4 | 3 | GA | EL,AL,FT,GW |
| 2.4 | 4 | 4 | 0 | PH,GW,GA,TGW | EL,AL,FT,GL |
| 2.5 | 1 | 6 | 1 | GA | EL,AL,FT,GL,GW,TGW |
| 2.6 | 3 | 3 | 2 | PH,GA,TGW | EL,AL,FT |
| 3.1 | 4 | 4 | 0 | PH,GW,GA,TGW | EL,AL,FT,GL |
| 3.2 | 1 | 5 | 2 | GA | EL,AL,FT,GL,GW |
| 3.3 | 3 | 3 | 2 | PH,GA,TGW | EL,AL,FT |
| 3.4 | 1 | 5 | 2 | GA | EL,AL,FT,GL,GW |
| 4.1 | 2 | 5 | 1 | GA,TGW | EL,AL,FT,GL,GW |

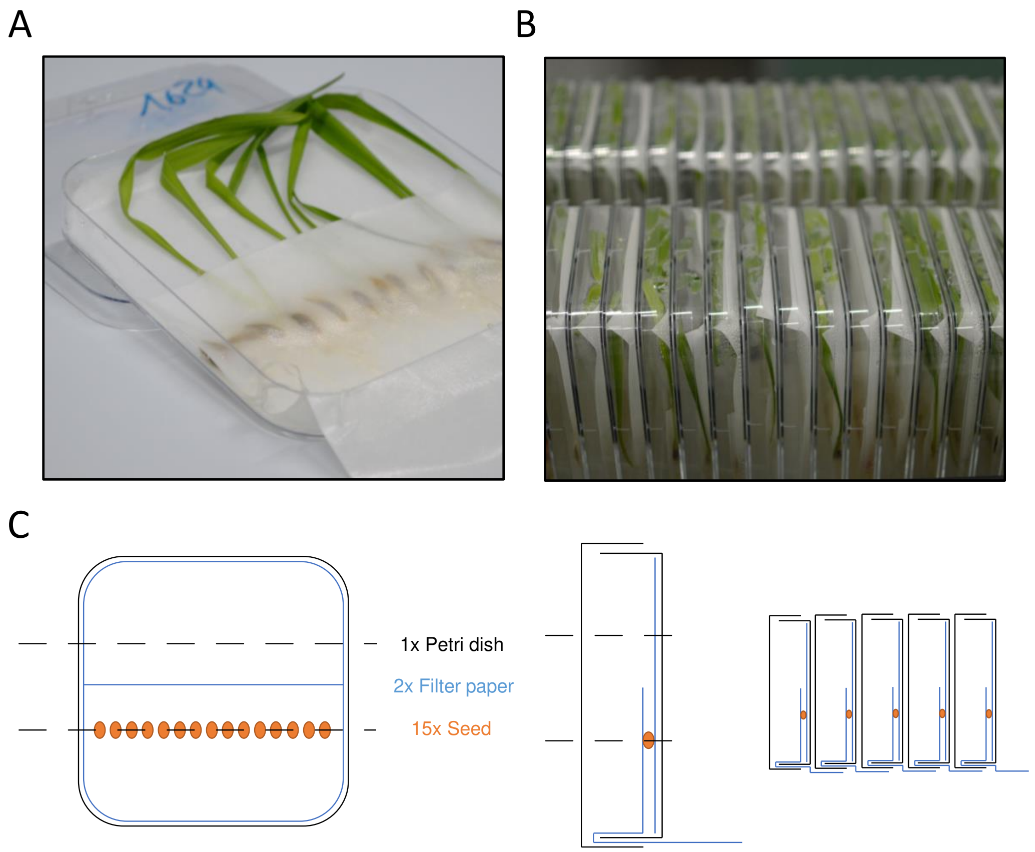

**Fig. S1.** Illustration of the cultivation system of (A) barley seedlings in Petri dishes using (B) space efficient cultivation. (C) Schematic overview of the approach used to cultivate barley seedlings in reach-in growth chambers.

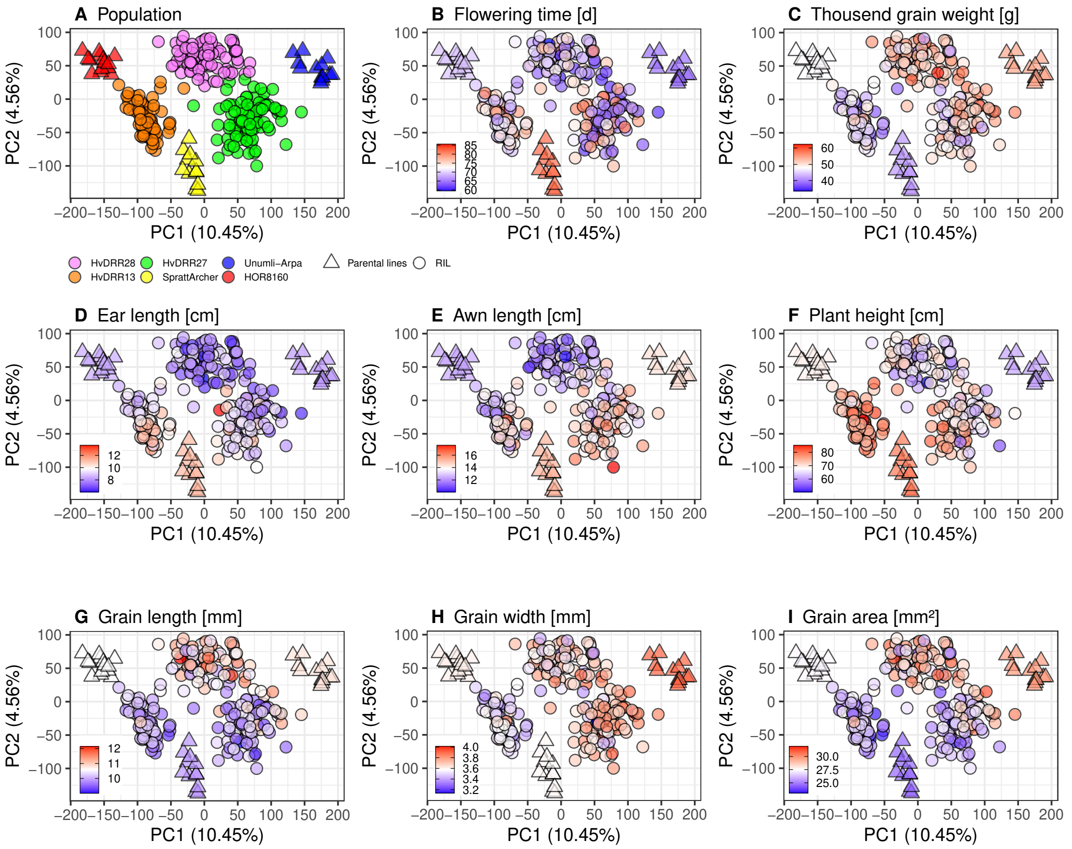

**Fig. S2.** Principal component analysis (PCA) based on $GE^{DEG}_{RNAseq}$, (A) overlayed with the information of the genetic material and (B-I) the adjusted entry means of all eight traits. Shape differentiate RIL and parental inbreds. PC 1 and PC 2 are the first and second principal component, respectively, and the number in parentheses refers to the proportion of variance explained by the principal components in percent.

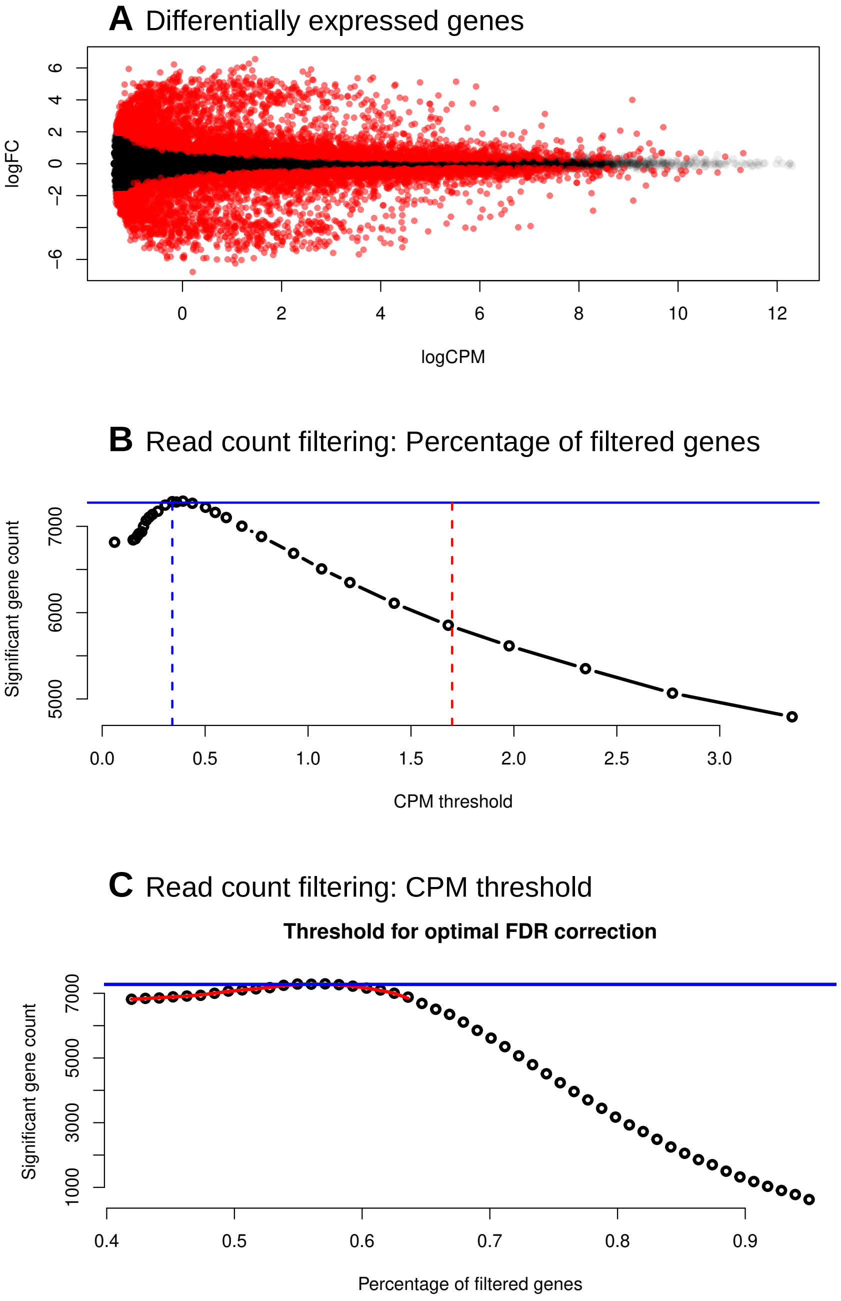

**Fig. S3.** overview of the read count filtering. (a) the number of differentially expressed genes shown in red could be maximized by adjusting the percentage of (b) filtered transcripts or (c) cpm threshold. the blue horizontal line indicating the maximum of differentially expressed genes counted. the dotted vertical lines show the chosen threshold (blue) and the edger standard threshold (red).

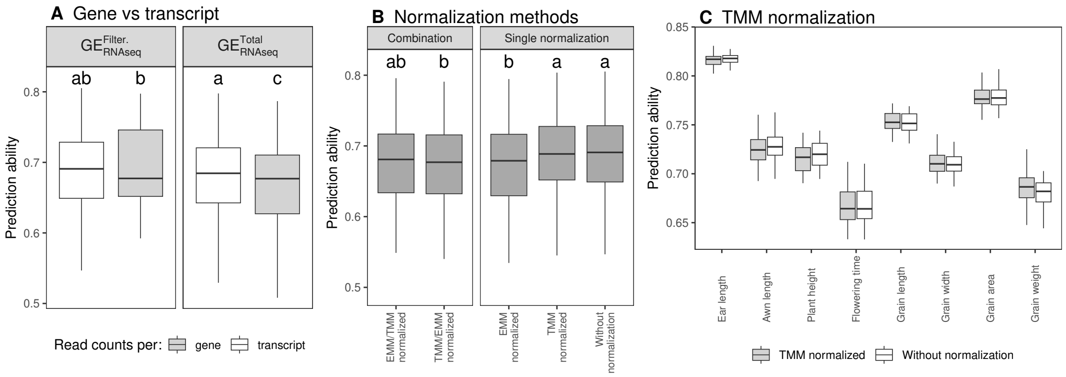

**Fig. S4.** Characterization of the expression data. (A) Comparison of the genomic prediction results for two different read counting methods: counting per gene and counting per transcript for $GE^{Total}_{RNAseq}$ and $GE^{Filter.}_{RNAseq}$. (B) Genomic prediction results for $GE^{DEG}_{RNAseq}$ compared based on the order of normalization: Trimmed Mean of the M-values (TMM) first or estimated marginal means (EMM) based on block effect first. (C) Genomic prediction abilities for $GE^{DEG}_{RNAseq}$ comparing unnormalized and TMM normalized data for all eight traits.

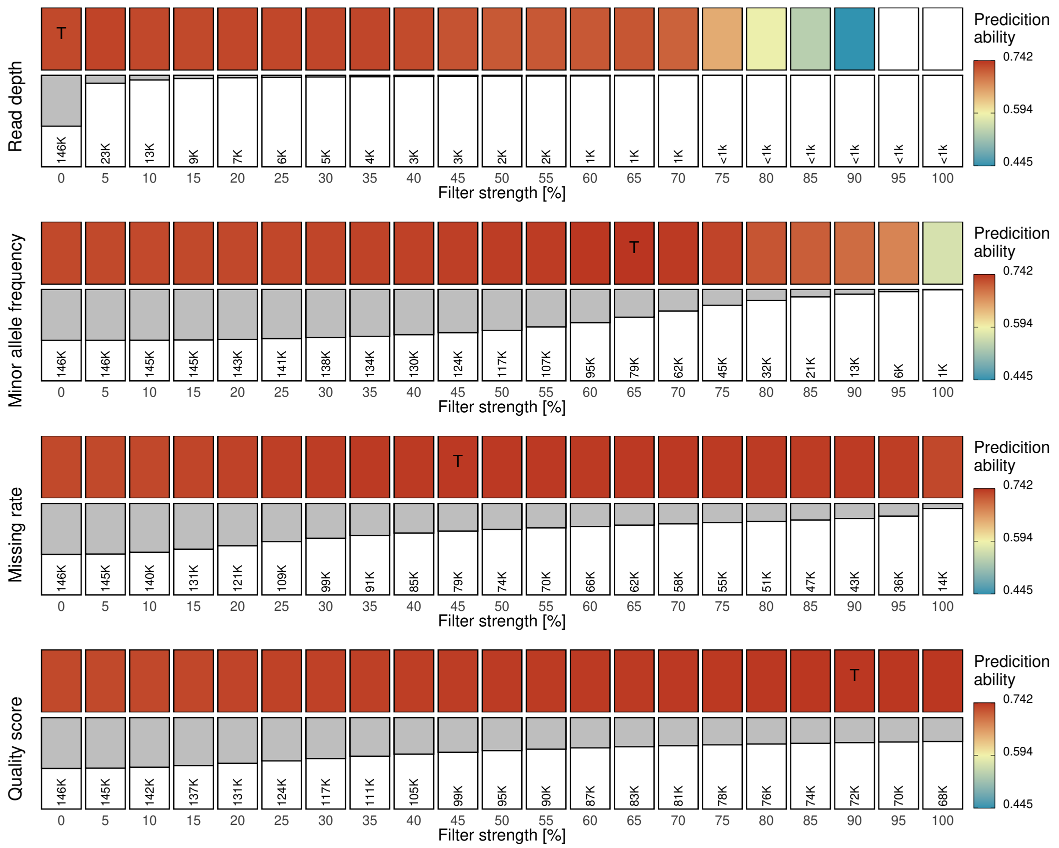

**Fig. S5.** Quality filtering of genomic variants of sequencing data based on genomic prediction (GP) performance. The performance shown are prediction abilities averaged across all eight traits. For each of the four criteria the prediction ability (top) and the remaining number of markers in thousands (bottom) are shown for 21 different relative filtering strength subsets from the minimum (0%) to the maximum (100%) value in the original dataset ($SNP^{Total}_{RNAseq}$). The best performing filter strength subset is marked (T). No GP was performed (white) when the number of remaining markers was insufficient.

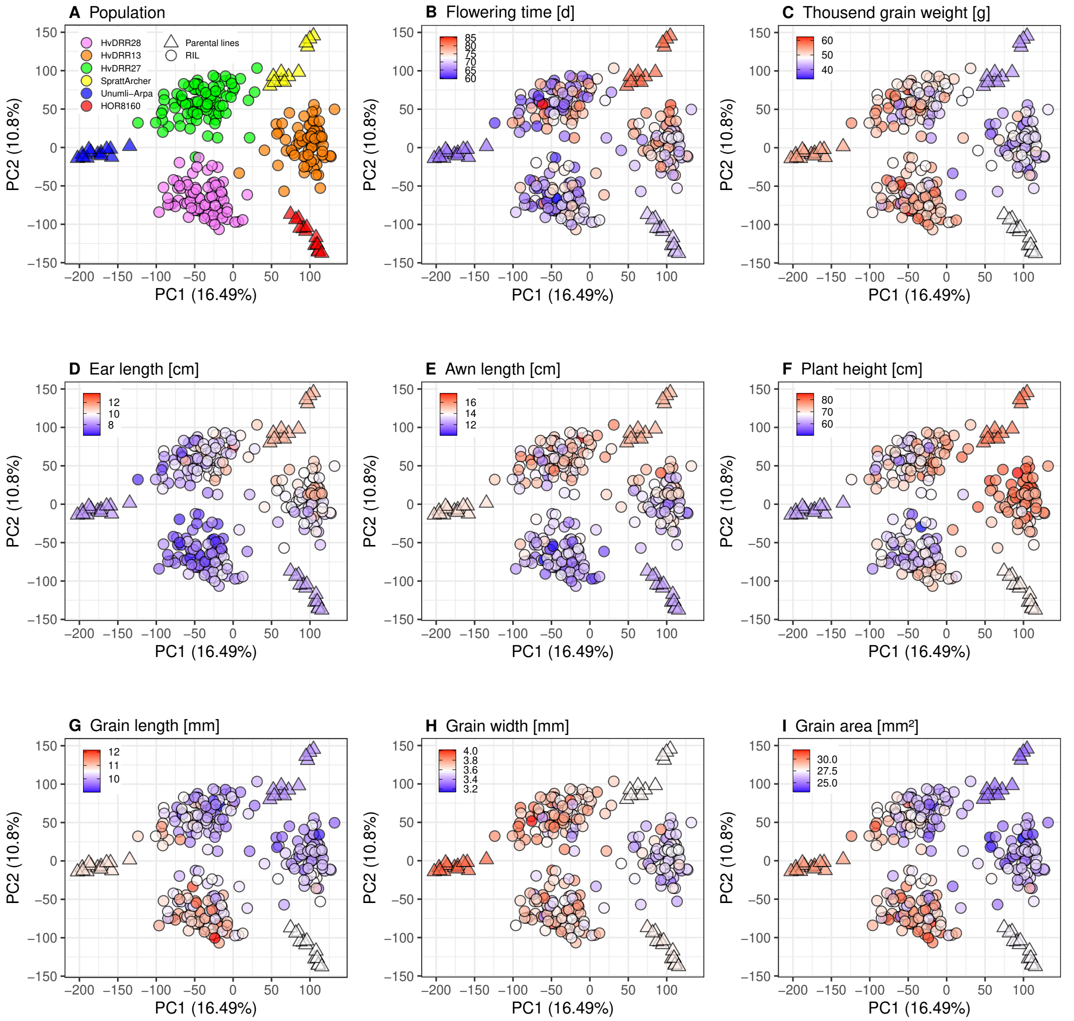

**Fig. S6.** Principal component analysis (PCA) based on $SNP^{QC}_{RNAseq}$, (A) overlayed with the information of the genetic material and (B-I) the adjusted entry means of all eight traits. Shape differentiate RIL and parental inbreds. PC 1 and PC 2 are the first and second principal component, respectively, and the number in parentheses refers to the proportion of variance explained by the principal components in percent.

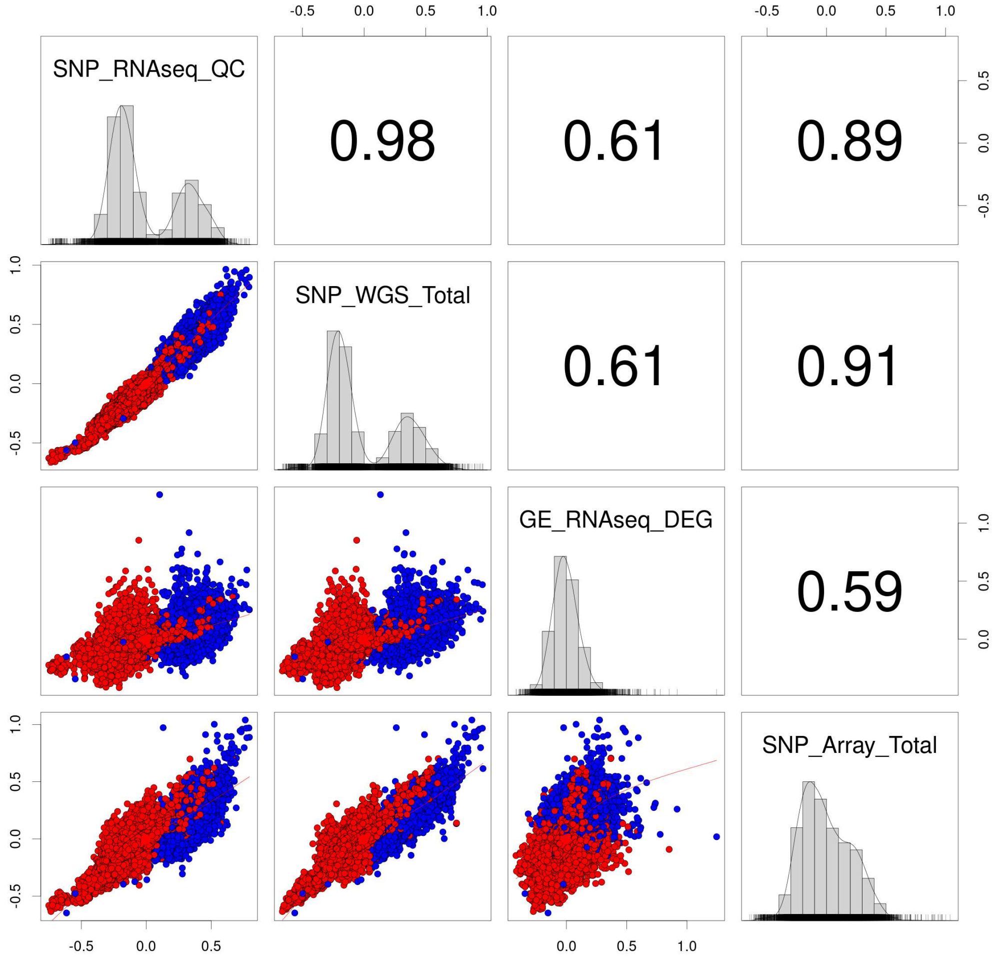

**Fig. S7.** Correlation plots of the additive relationship matrices of the datasets: $SNP^{QC}_{RNAseq}$ (SNP_RNAseq_QC), $SNP^{Total}_{WGS}$ (SNP_WGS_Total), $GE^{DEG}_{RNAseq}$ (GE_RNAseq_DEG), $SNP^{Total}_{Array}$ (SNP_Array_Total). The dots are colored by sample relationship, with intra-population comparisons blue and inter-population comparisons red.

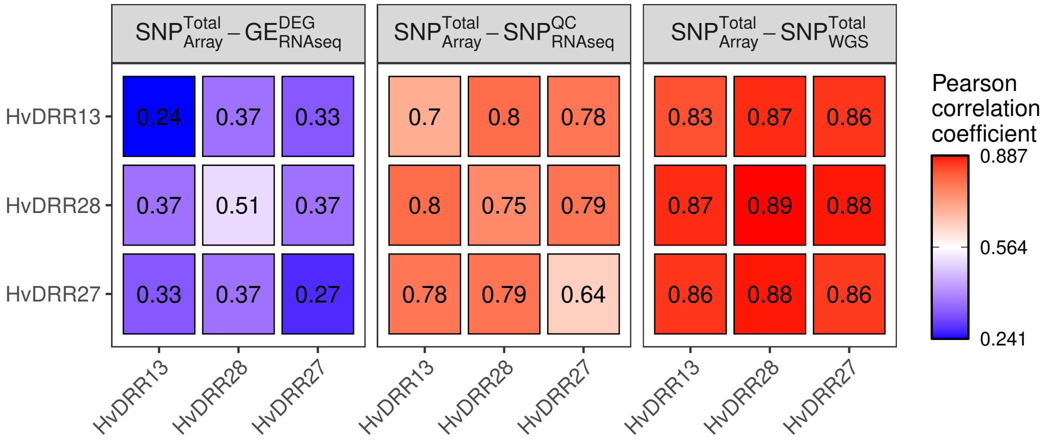

**Fig. S8.** Intra and Inter-population additive relationship matrices comparison. Heat map of Pearson correlation coefficients between the covariances of population segments of two additive relationship matrices. Comparison between $SNP_{Array}^{Total}$ and the three remaining main datasets: $SNP_{RNAseq}^{QC}$, $SNP_{WGS}^{Total}$, and $GE_{RNAseq}^{DEG}$.

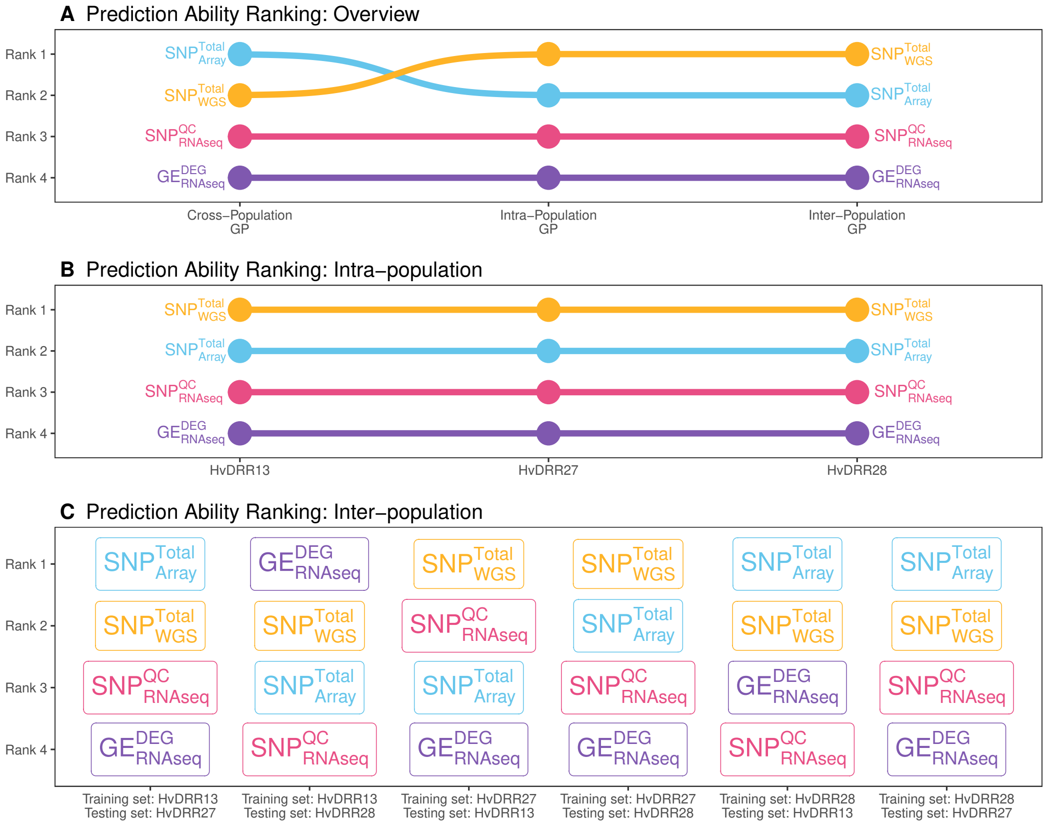

**Fig. S9.** Prediction Ability (PA) ranking for (A) intra-population and (B) inter-population comparisons using the best performing datasets from each data origin group.The training set size was set to 50 for all tests.

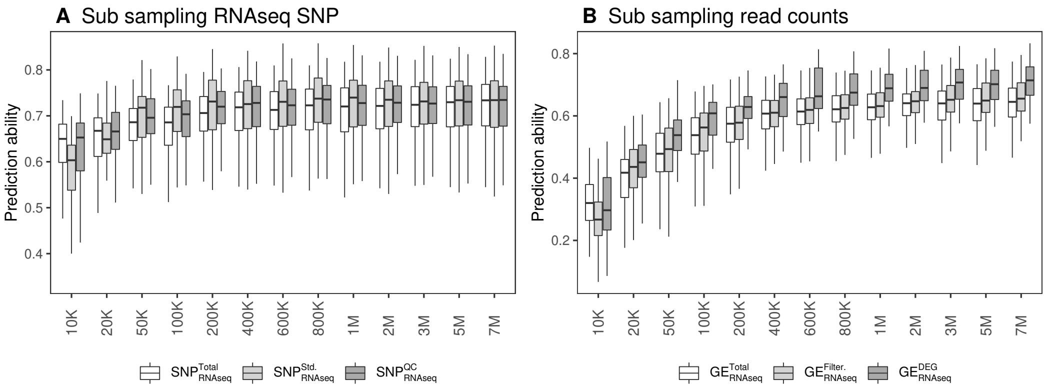

**Fig. S10.** Genomic prediction (GP) performance of artificially reduced sequencing depth subsets created by random read sub-sampling of (A) RNA-Seq genomic variant datasets ($SNP^{Total}_{RNAseq}$, $SNP^{QC}_{RNAseq}$) and (B) gene expression datasets ($GE^{Total}_{RNAseq}$, $GE^{Filter.}_{RNAseq}$, $GE^{DEG}_{RNAseq}$) including 155 of the 240 samples. The sub-sampling ranged in 13 steps between 10 thousand to 7 million reads.

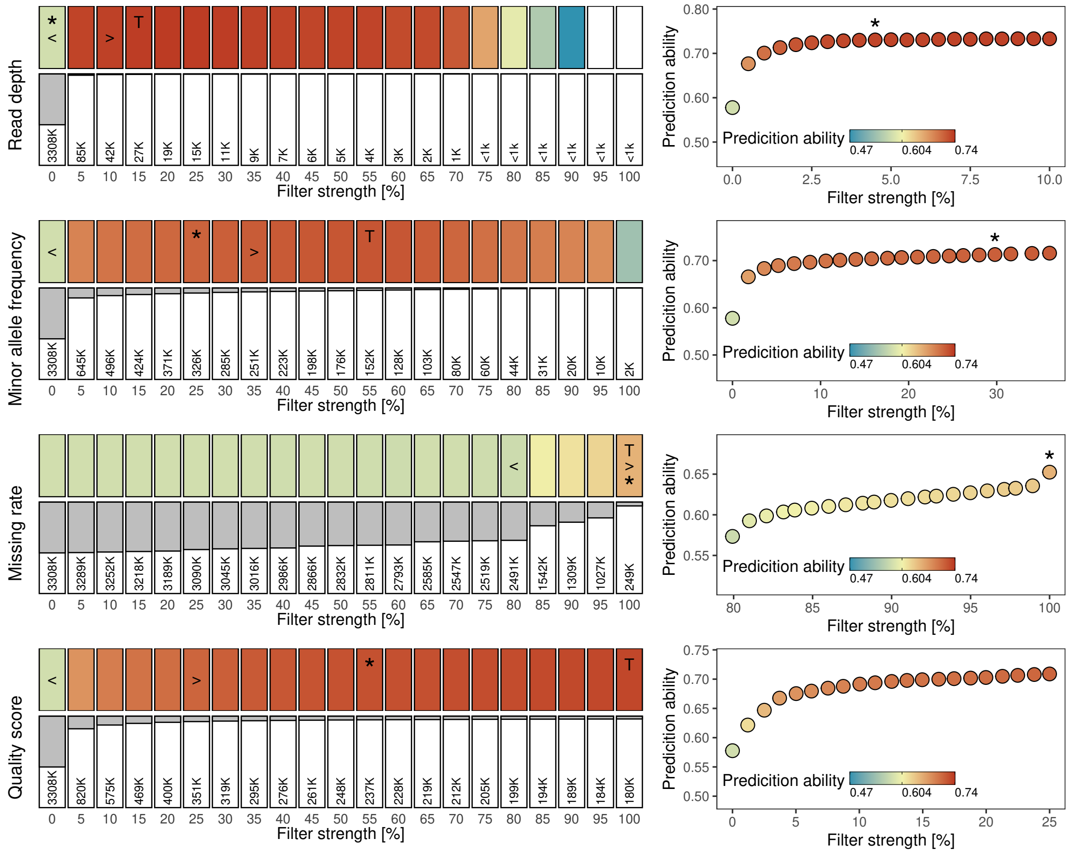

**Fig. S11.** Quality filtering of genomic variants of sequencing data based on genomic prediction (GP) performance. Quality filtering is an essential step in maximizing the GP potential and can have a larger effect on the results. We tested the impact of quality filtering on a less strictly cleaned dataset by creating $SNP^{Raw}_{RNAseq}$, an alternative version of $SNP^{Total}_{RNAseq}$, which included heterozygous / inconsistent allele calls and marker with missing parental data. From the 5.9M raw variants $SNP^{Raw}_{RNAseq}$ included 3.4M, which is much more then $SNP^{Total}_{RNAseq}$ (148K) and it resulted in a reduction GP performance by 0.15, but applying the same quality filtering workflow resulted in a subset with a comparable GP performance to $SNP^{QC}_{RNAseq}$.

The performance shown are prediction abilities averaged across all eight traits. For each of the four criteria the prediction ability (left, top) and the remaining marker in thousands (left, bottom) are shown for 21 different relative filtering strength subsets from the minimum (0%) to the maximum (100%) value in the original dataset (SNPTotal RNAseq). The best performing filter strength subset is marked top (T) as well as the optimal filter strength subset (*). The filtering subset marked * are not significantly ($p > 0.05$) different to T. The filter strength region with the highest impact on GP performance is shown in detail (right). The section is outlined on the overview as from start (<) to end (>).
